# Acute Tumor Transition Angle on Computed Tomography Predicts Chromosomal Instability Status of Primary Gastric Cancer: Radiogenomics Analysis from TCGA and Independent Validation

**DOI:** 10.3390/cancers11050641

**Published:** 2019-05-09

**Authors:** Ying-Chieh Lai, Ta-Sen Yeh, Ren-Chin Wu, Cheng-Kun Tsai, Lan-Yan Yang, Gigin Lin, Michael D. Kuo

**Affiliations:** 1 Department of Medical Imaging and Intervention, Chang Gung Memorial Hospital, Chang Gung University, Taoyuan 333, Taiwan; cappolya@gmail.com; 2 Imaging Core Lab, Institute for Radiological Research, Chang Gung Memorial Hospital, Chang Gung University, Taoyuan 333, Taiwan; klem.tsaick@gmail.com; 3 Department of General Surgery, Chang Gung Memorial Hospital, Chang Gung University, Taoyuan 333, Taiwan; tsy471027@cgmh.org.tw; 4 Department of Pathology, Chang Gung Memorial Hospital, Chang Gung University, Taoyuan 333, Taiwan; renchin.wu@gmail.com; 5 Clinical Metabolomics Core Lab, Chang Gung Memorial Hospital, Taoyuan 333, Taiwan; 6 Clinical Trial Center, Chang Gung Memorial Hospital, Taoyuan 333, Taiwan; lyyang0111@gmail.com; 7 Department of Diagnostic Radiology, University of Hong Kong, Hong Kong 999077, China

**Keywords:** chromosomal instability, computed tomography, gastric cancer, morphology, radiogenomics

## Abstract

Chromosomal instability (CIN) of gastric cancer is correlated with distinct outcomes. This study aimed to investigate the role of computed tomography (CT) imaging traits in predicting the CIN status of gastric cancer. We screened 443 patients in the Cancer Genome Atlas gastric cancer cohort to filter 40 patients with complete CT imaging and genomic data as the training cohort. CT imaging traits were subjected to logistic regression to select independent predictors for the CIN status. For the validation cohort, we prospectively enrolled 18 gastric cancer patients for CT and tumor genomic analysis. The imaging predictors were tested in the validation cohort using receiver operating characteristic curve (ROC) analysis. Thirty patients (75%) in the training cohort and 9 patients (50%) in the validation cohort had CIN subtype gastric cancers. Smaller tumor diameter (*p* = 0.017) and acute tumor transition angle (*p* = 0.045) independently predict CIN status in the training cohort. In the validation cohort, acute tumor transition angle demonstrated the highest accuracy, sensitivity, and specificity of 88.9%, 88.9%, and 88.9%, respectively, and areas under ROC curve of 0.89. In conclusion, this pilot study showed acute tumor transition angle on CT images may predict the CIN status of gastric cancer.

## 1. Introduction

Gastric cancer is one of the most common and aggressive solid malignancies worldwide, with the highest incidence in Asia [1,2]. Patients undergoing standard treatment (i.e., surgical resection plus adjuvant chemotherapy or radiochemotherapy) have high rates of tumor recurrence (20%–40%) [3,4]. The heterogeneity in clinical outcomes in gastric cancer is consistent with other solid tumors, and genomic analysis has repeatedly shown that tumors are molecularly diverse [5]. Therapies specifically targeting key molecular features can have clinical outcomes beyond those of traditional standard therapies [5,6]. Conventional Lauren [7] and World Health Organization [8] classifications are based on histopathologic features and have limited implications in guiding personalized therapy for gastric cancer patients [9]. More recently, the Cancer Genome Atlas (TCGA) research group developed a molecular classification system based on gene expression profiling for gastric cancer. It emphasizes a molecular pathogenesis perspective, providing a potential roadmap for targeted therapy [9]. Among the four TCGA subtypes—Epstein–Barr virus (EBV)-positive, microsatellite unstable, chromosomal instability (CIN), and genomically stable—CIN subtype gastric cancer accounts for nearly half of all gastric cancer cases [9]. The CIN status is defined as a high degree of somatic copy number variation by gaining or losing chromosomes [10]. Traditionally, the CIN status of tumors are detected by molecular cytogenetic techniques such as comparative genomic hybridization, polymerase chain reaction, flow cytometry, or single nucleotide polymorphism arrays-based methods [11]. In daily clinical practice, these complex genomic analysis techniques may not provide timely information for decision making in cancer treatment, and tumor specimen is not always available. Besides, CIN is a complex, heterogenous, and ongoing process that initiates and drives oncogenesis, and the profile of CIN may not be completely delineated by traditional methods. If CIN subtype gastric cancer could be predicted using clinical, imaging, or histopathologic data collected in the routine evaluation and work-up of gastric cancer patients, they may provide rapid and complementary information before genomic analysis results [12].

Radiogenomics (the science of multiscale data fusion) is a powerful, robust, and scalable tool that has been applied across different tumor types and imaging modalities to address many crucial questions in oncology [13]. It has been used to create “association maps” between large-scale multilevel genomic data and image features from clinical imaging to identify clinically significant prognostic and predictive biomarkers. It has also been used to define molecular patterns associated with particular image phenotypes in different imaging modalities and tumor types. Radiogenomics links and validates associations between imaging signatures, clinical findings, and molecular pathogenesis [14]. Computed tomography (CT) is a routine preoperative evaluation modality in gastric cancer patients. With the development of isotropic imaging and multiplanar reconstruction, early gastric cancer can now be detected through multidetector CT, with a reported detection rate of 90% [15]. The TNM staging system is a widely used cancer staging system based on the tumor extent, the lymph node spread, and the presence of metastasis [16]. Studies have mainly focused on the key components of the TNM staging system to improve diagnostic accuracy. Little attention has been paid to other imaging traits such as tumor morphology, texture, or contrast enhancement pattern, which do not contribute to the TNM staging system. Through radiogenomic analysis, imaging traits that provide additional information can be extracted during routine imaging examination without additional costs. No study has investigated the radiological phenotypes associated with clinically significant genomic signatures in gastric cancer. Whether radiogenomic features from CT imaging can be used to identify CIN subtype gastric cancer remains a critical and unaddressed question.

In this study, we aimed to investigate the role of CT imaging traits in predicting the CIN status of gastric cancer.

## 2. Results

### 2.1. Training Cohort and Imaging Predictors

We screened 443 patients in the TCGA gastric cancer cohort and selected 43 patients with complete CT imaging, genomic, and clinical data. We further excluded three patients from the training cohort: two for small tumor diameter (<1 cm) and one for predominant distal esophageal tumor. Thus, the final training cohort comprised 40 patients: 35 men and 5 women (Table 1). The median age was 68 years (range, 36–79). The median tumor diameter was 5.4 cm (range, 2.8–12.5). None of the patients had early gastric cancer, defined as tumor limited to the mucosa or submucosa regardless of lymph node status, according to the Japanese Gastric Cancer Association [17]. Of the 40 patients, 30 (75%) had CIN subtype gastric cancer, and the other 10 (25%) had non-CIN subtype gastric cancer (2 EBV-positive, 5 microsatellite unstable, and 3 genomically stable subtype). Of the 30 CIN subtype gastric cancers, 21 tumors (70%) were categorized as Borrmann type I or II in tumor shape. No significant association was found between the Borrmann classification and CIN status (*p* = 0.135). From the training cohort, two CT imaging traits independently predicted the CIN status of gastric cancer: smaller tumor diameter (odds radio [OR]: 0.54, *p* = 0.017) and acute tumor transition angle (OR: 7.41, *p* = 0.045) (Table 2).

### 2.2. Validation Cohort

**Figure 1:**
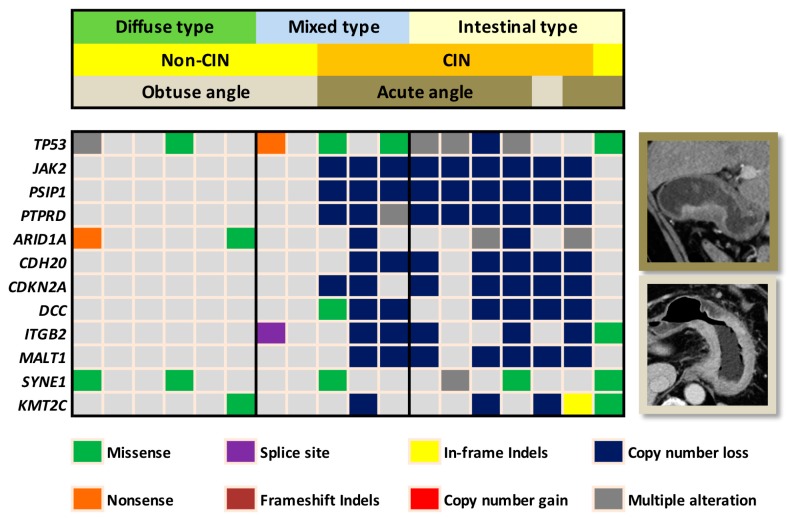
Heatmap demonstrates the correlation of the chromosomal instability (CIN) status and tumor transition angle on computed tomography (CT) in the validation cohort. Eight out of the 9 non-CIN gastric cancers presented with obtuse angle whereas 8 out of the 9 CIN gastric cancers presented with acute angle on CT. In the Lauren mixed type tumors, transition angle on CT clearly defined the CIN versus non-CIN status. For the interest of space, only the leading 12 out of the 409 gene mutations were demonstrated on the heatmap.

The validation cohort included 18 patients, consisting of 11 men and 7 women (Table 1). The median age was 68 years (range, 47–87). The median tumor diameter was 3.7 cm (range, 1.7–11.6). The tumor diameter of the validation cohort was significantly smaller than that of training cohort (*p* = 0.01). No significant differences were observed in TNM staging between the validation and training cohorts. Using the Lauren system, the histologic type of gastric cancer was classified as intestinal type in 7, diffuse type in 6, and mixed type in 5 patients. In the validation cohort, 9 patients had CIN subtype gastric cancer (Figure 1). The CIN subtype gastric cancer was predominantly of the Lauren intestinal type, and the non-CIN subtype gastric cancer was predominantly of the Lauren diffuse type. Variance in the degree of aneuploidy (i.e., copy number loss or gain) was observed in the CIN subtype gastric cancers. The median number of aneuploidy genes was 63 (range, 25 to 200).

### 2.3. Diagnostic Accuracy of Imaging Predictors

**Figure 2:**
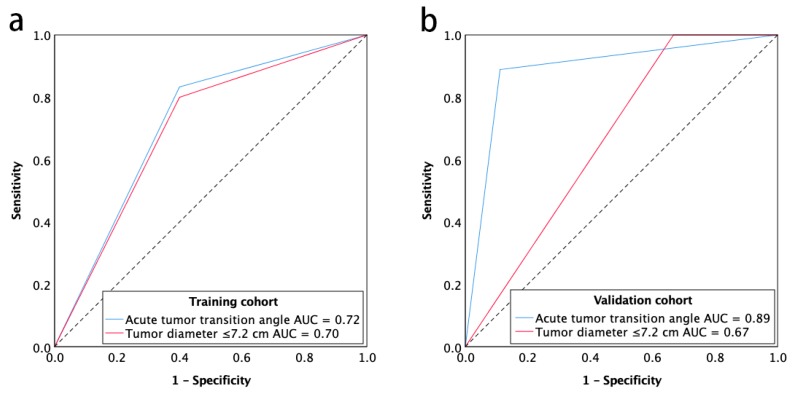
Receiver operating characteristic curve for the chromosomal instability status of gastric cancer with acute tumor transition angle and tumor diameter ≤7.2 cm for training cohort (**a**) and validation cohort (**b**). Note—AUC, areas under the receiver operating characteristics curve.

**Figure 3:**
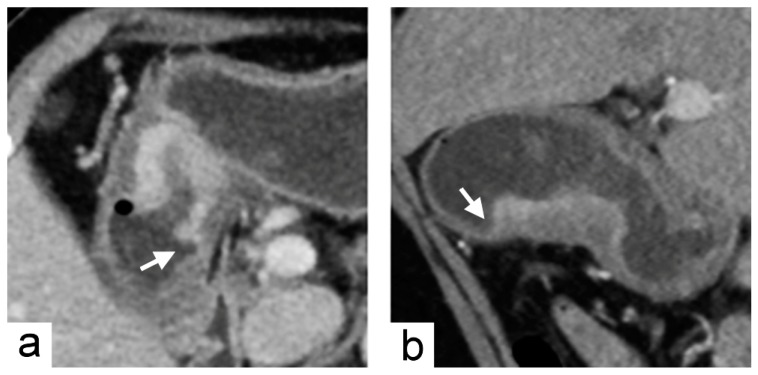
Chromosomal instability subtype gastric cancer in a 79-year-old female who underwent preoperative contrast-enhanced computed tomography. (**a**) portal venous phase axial and (**b**) arterial phase sagittal images showed focal wall thickening of the greater curvature side of the stomach with the largest diameter of 3.1 cm. In the imaging traits evaluation, the tumor morphology was defined to be acute tumor transition angle (arrows in (**a**,**b**)), well-defined margin and polypoid shape.

**Figure 4:**
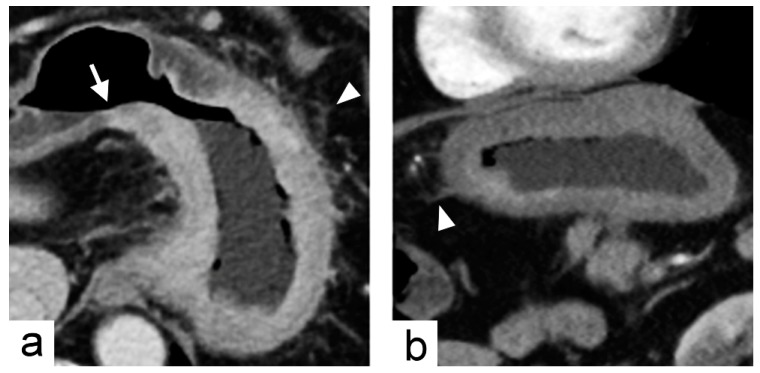
Non-chromosomal instability subtype gastric cancer in a 68-year-old male who underwent preoperative contrast-enhanced computed tomography. (**a**) portal venous phase axial and (**b**) arterial phase sagittal images showed extensive circumferential wall thickening involving the gastric fundus, cardia and body with the largest diameter of 6.6 cm. The tumor had obtuse tumor transition angle (arrow in (**a**)), ill-defined margin and infiltrative shape in morphology. Because of the peri-gastric stranding densities, the lesion was also defined to have “serosal invasion” imaging trait (arrowheads in (**a**,**b**)), which was later confirmed by histopathology analysis.

The imaging predictors identified from the training cohort were tested in the validation cohort. A tumor diameter cutoff value of ≤7.2 cm was obtained from the training cohort using receiver operating characteristic curve (ROC) analysis. The diagnostic accuracy of imaging predictors was evaluated by ROC analysis in the validation cohort. The areas under the receiver operating characteristic curve (AUC) were 0.89 (95% CI, 0.72–1.00) for acute tumor transition angle and 0.67 (95% CI, 0.41–0.93) for tumor diameter ≤7.2 cm in the validation cohort (Figure 2). As the more accurate imaging predictor of the CIN status of gastric cancer, acute tumor transition angle achieved an accuracy, sensitivity, and specificity of 88.9%, 88.9%, and 88.9% in the validation cohort as detailed in Table 3. Examples of imaging traits analysis of CIN and non-CIN gastric cancers are demonstrated in Figure 3 and Figure 4, respectively.

## 3. Discussion

Our study showed that the two imaging traits—smaller tumor diameter and acute tumor transition angle—independently predicted the CIN status of gastric cancer in the training cohort. In the independent validation cohort, the imaging trait of acute tumor transition angle was the more accurate imaging predictor, with sensitivity and specificity of 88.9% and 88.9%, respectively; this trait may noninvasively predict the CIN status of gastric cancer. Consistent with the findings for our prospective validation cohort, CIN subtype gastric cancer accounted for approximately 50% of the study cohort of TCGA classification study [9]. The CIN subtype has better responses to adjuvant chemotherapy, whereas the microsatellite unstable and genomically stable subtype have only moderate benefits and no benefits from adjuvant chemotherapy, respectively [18]. By knowing the CIN status of gastric cancer, a personalized adjuvant treatment strategy including conventional chemotherapy and target therapy could potentially be tailored for gastric cancer patients based on their radiogenomic CT profile.

The Borrmann classification system is a morphologic classification of advanced gastric cancer based on endoscopy or macroscopic pathology examination; it provides a simple and valuable prediction of lymph node metastasis and survival [19,20]. The CIN subtype gastric cancers in the present study were predominantly of the intestinal type according to Lauren classification, which is in line with the literature data [9]. Although the Lauren intestinal type had been reported to be associated with less advanced Borrmann morphology (i.e., more likely acute tumor transition angle) [9,21], the association of Borrmann classification with CIN subtype gastric cancer could not be demonstrated in our study. Besides, it remains unaddressed whether the transition angle changes as the tumor grows, because our training and validation cohorts comprised mostly advanced gastric cancers. The only one CIN subtype gastric cancer showing obtuse tumor transition angle had a relatively low number of aneuploidy genes of 34. However, we could not exclude the patient from the validation cohort because the patient was not an outliner in degree of aneuploidy. The relationship between the degree of aneuploidy and tumor transition angle was undetermined due to limited sample size of this study. On the other hand, the Lauren diffuse type gastric cancer is enriched in the genomically stable subtype gastric cancer of TCGA classification system [9]. Zhou et al. had demonstrated that abnormal expression of E-cadherin, which is a major adhesion molecule in the cell-cell junction, correlated with the Lauren diffuse type gastric cancer and more infiltrative morphology (Borrmann type III and IV) [22].

EBV-associated gastric cancers account for approximately 9% of all gastric cancers [23]. They are characterized by high EBV burden and DNA promoter hypermethylation [9]. A study of 10 EBV-associated gastric cancer patients showed that location in the upper gastric region, large thickness-to-width ratio, or bulky mass projecting from the wall were CT features of EBV-associated gastric cancer [24]. In our TCGA training cohort, two patients had EBV-positive subtype gastric cancer, and both of the tumors were located in the upper gastric body. However, further CT imaging feature analysis of EBV-positive subtype gastric cancer was precluded due to limited patient number.

To accurately extract imaging traits from CT images, the patients with smaller tumor diameter (<1 cm) were excluded from this study, which may account for the relatively large cutoff value of 7.2 cm obtained from the training cohort by ROC analysis. The diagnostic accuracy was unsatisfactory based on tumor diameter in the validation cohort, plausibly because of the significant difference in tumor diameter between the training and validation cohort. Future study of larger sample size may provide a more optimal cutoff value for tumor diameter in predicting the CIN status of gastric cancer.

Tsurumaru et al. demonstrated the association between gastric cancer histopathologic types and the contrast enhancement pattern on dynamic contrast-enhanced CT images [25,26]. In our study, no association was observed between the double-layered enhancement pattern and CIN status. Consistent with our result, Lauren diffuse type gastric cancer (usually non-CIN subtype gastric cancer) frequently showed a double-layered pattern on arterial phase images and a single-layered pattern on delayed phase images [25].

The novel concept of radiogenomics provides a connection between imaging traits and genetic information of cancers [13]. In the era of precision medicine, there is an increasing need to classify and treat cancers on a molecular basis because the clinical outcomes and treatment response may vary even if the cancers are histologically similar [27]. Although the advancement of high-throughput analysis has facilitated more rapid and lower cost genomics data acquirement [14], the inherent limitation is three-fold. First, genomic profiles require adequate tumor tissue, specialized equipment, and technical expertise. Second, surgical or image-guided tumor biopsy is not always feasible in cancer patients due to risks and possible complications associated with the biopsy procedure. Third and most importantly, a tumor may have different internal components with distinct gene expression patterns (i.e., intratumoral heterogeneity) [28]. Thus, tumor specimens only represent a small portion of the tumor rather than the whole tumor. Noninvasive imaging examinations, which are routinely performed for clinical staging, have the potential to provide overall perspectives of the tumor and demonstrate intratumoral heterogeneity. It will be helpful if we can correlate imaging traits (i.e., imaging phenotype or radiophenotype) to certain genetic subtypes or gene expression patterns of cancers [29]. Radiogenomic features have been demonstrated to be associated with the luminal B subtype of breast cancer [30] or the VHL gene mutation in clear cell renal cell carcinoma [31].

Serum biomarker is another potential approach to predict the CIN status of gastric cancer. CIN in plasma or serum cell-free DNA has been used to detect ovarian cancer or prostate cancer [32,33]. In gastric cancer, a study had demonstrated plasma DNA concentration as diagnostic biomarker by quantifying plasma cell-free DNA [34]. However, it is still unclear whether CIN in serum cell-free DNA correlates with the CIN subtype gastric cancer of TCGA classification, and it may be a feature direction of research. The potential serum biomarker may be a complement to our CT imaging predictor and enhance the accuracy in predicting CIN subtype gastric cancer.

This study has several limitations. First, although a prospective validation cohort was included, a retrospective cohort was used for training, and both cohorts have small sample sizes. The preliminary results of this study warrant further validation in a larger gastric cancer cohort with surrogate profiles of CIN, such as an immunohistochemistry panel of MLH1, p53, and EBER staining [35], or CIN70 signature—70 genes that correlate with high levels of aneuploidy [36,37]. Second, the majority of enrolled patients had resectable advanced gastric cancers in the current report. Further study should include more patients with more unresectable tumors or early gastric cancers, or even in CIN animal models, to test the generalizability of this pilot finding. Third, we determined tumor morphology on multiplanar reconstruction images. Although these images allow radiologists to evaluate target lesions in different orientations, the partial volume effect may still lead to the incorrect interpretation of morphologic imaging traits. Future CT studies with three-dimensional reformatted virtual gastroscopy may provide more precise and global views of gastric cancer when interpreting imaging traits. Lastly, although CT texture features might correlate with immunochemical biomarkers such as E-cadherin, Ki67, VEGFR2, and EGFR in gastric cancer [38], the large feature numbers might cause false-discovery in our limited sample sizes. Nevertheless, our initial report is the first study utilizing the radiogenomic approach to analyze the molecular subtype of gastric cancer. Radiogenomic analysis of gastric cancer including the analysis of CT texture features may be a future research direction.

## 4. Materials and Methods

### 4.1. Study Patients and Data Collection

This study was designed as a disease landscape study with no prespecified hypothesis. The institutional review board approved the protocol of this study (project number: 201601916B0C601), which had both retrospective and prospective components. A waiver of consent was obtained for the retrospective phase, which involved the extraction of imaging traits from a publicly available database. For the prospective phase, informed consent was obtained from participants in a tertiary referral center. For patient enrolment, a dedicated gastric cancer interdisciplinary team screened patents through image examination and molecular analysis of tissue specimens, as described herein. 

### 4.2. Training Cohort

The public data portal of the TCGA provides public data of a cohort of gastric cancer patients containing complete genomic sequencing and clinical data (https://cancergenome.nih.gov). The Cancer Imaging Archive data set contains publicly available CT images of a subset of TCGA cohort patients (http://www.cancerimagingarchive.net). We selected gastric cancer patients with preoperative CT images from the TCGA cohort as the training cohort. To accurately extract imaging traits from CT images, the patients with tumor diameter <1 cm on CT images were excluded.

### 4.3. Imaging Traits Evaluation

**Figure 5:**
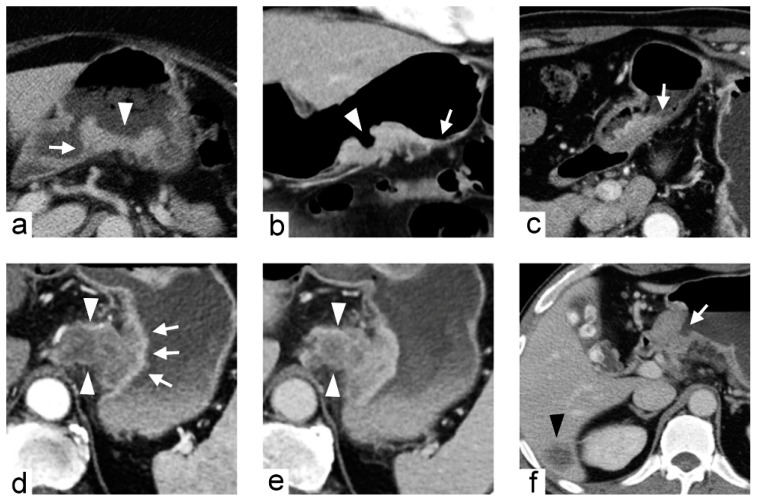
Imaging traits of gastric cancers demonstrated in different patients. (**a**) 71-year-old female with gastric cancer at posterior wall of gastric body. Contrast-enhanced computed tomography (CT) during portal venous phase axial image demonstrates the tumor with fungating shape (arrowhead), well-defined margin and acute tumor transition angle (arrow). (**b**) 61-year-old female with gastric cancer at greater curvature of gastric body. Contrast-enhanced CT during arterial phase coronal image demonstrates the tumor with ulcerative shape (arrowhead), ill-defined margin and obtuse tumor transition angle (arrow). (**c**) 66-year-old male with gastric cancer at antrum. Contrast-enhanced CT during arterial phase axial image demonstrates the tumor with polypoid shape, ill-defined margin and obtuse tumor transition angle (arrow). (**d**,**e**) 73-year-old male with gastric cancer at cardia. Contrast-enhanced CT during arterial phase (**d**) and portal venous phase (**e**) axial images demonstrate the tumor with an inner layer of higher contrast enhancement (arrows in (**d**)) and an outer extra-gastric portion of heterogeneously lower contrast enhancement (arrowheads in (**d**,**e**)). (**f**) 56-year-old male with gastric cancer at antrum. Contrast-enhanced CT during arterial phase axial image demonstrates the tumor with luminal obstruction (arrow) and liver metastasis (arrowhead).

Fourteen qualitative and two quantitative imaging traits were defined and analyzed for their association with the CIN status of gastric cancer (Table 4 and Figure 5). The “tumor shape” imaging trait was defined according to the Borrmann classification system (a morphologic classification of advanced gastric cancer) [20]. Two radiologists (Ying-Chieh Lai and Gigin Lin with 3 and 12 years of oncology imaging experience, respectively) independently reviewed CT images of all patients and were blinded to their clinical variables and genomic analysis results. Imaging traits extracted from CT images were evaluated on the picture archiving and communication system. In case of discrepancy in interpretation, the final results of imaging traits were based on a consensus between the two radiologists.

### 4.4. Validation Cohort

From May 2016–April 2017, consecutive gastric cancer patients from our center were screened and included in the validation cohort. The inclusion criteria were (1) age of 20–80 years; (2) histologically confirmed adenocarcinoma of the stomach; and (3) tumors considered resectable by gastric cancer interdisciplinary team. The exclusion criteria were (1) tumor diameter <1 cm on CT images; (2) prior gastric surgery; and (3) prior neoadjuvant chemotherapy or chemoradiotherapy. The patients underwent preoperative CT images within 14 days of surgery in accordance with the institutional CT protocol for gastric cancer. Histopathologic and genomic analysis of the gastric cancer specimens obtained via surgical resection was performed.

### 4.5. Imaging Analysis

All CT examinations of validation cohort were performed using 320-detector row CT (Aquilion ONE; Toshiba Medical Systems, Otawara, Japan). Oral contrast medium of 500 mL water was administered before imaging to distend the stomach, and intravenous contrast medium of 100 mL iohexol (350 mg iodine per millilitre, Omnipaque 350; GE Healthcare, Princeton, NJ, USA) was administrated using a power injector, with an injection rate of 3 mL/s. Multiphase (arterial and portal venous phases) contrast-enhanced CT imaging was performed as per the institutional standard CT protocol for gastric cancer. Multiphase CT imaging was performed after an empirical delay from initiation of contrast medium injection. The delay time was 25 and 70 s for arterial and portal venous phase imaging, respectively. The arterial phase scan focused on the stomach, and portal venous phase imaging was performed from the abdomen to pelvis. Coronal and sagittal multiplanar reconstruction images were used for more precise tumor detection and invasion depth evaluation. CT scan parameters were as follows: 120 kVp and automatic tube current modulation and image reconstruction to 5-mm thickness and at 5-mm intervals for viewing on a picture archiving and communication system. Two radiologists (Ying-Chieh Lai and Gigin Lin) who were blinded to the genomic analysis results independently tested predictors of CIN subtype gastric cancer on the validation cohort. The final results were based on a consensus between the two radiologists.

### 4.6. Histopathologic and Genomic Analysis

A gastrointestinal pathology specialist (Ren-Chin Wu, 12 years of experience) evaluated all haematoxylin and eosin-stained tissue slides of the validation cohort and provided information on the Lauren histologic type and the local invasion and lymph node metastasis status. Patients were classified to CIN subtype and non-CIN subtype according to the TCGA system [9], independent to the clinical information and CT imaging results. Genomic DNA was extracted from Formalin-fixed paraffin-embedded (FFPE) tumor samples using the QIAamp DNA FFPE Tissue Kit (Qiagen, Hilden, Germany); DNA was quantified using the Quant-iT dsDNA HS Assay (Invitrogen, Waltham, MA, USA). Genomic DNA (80 ng) was amplified using four pools of 15,992 primer pairs (Ion AmpliSeq Comprehensive Cancer Panel, Life Technologies, Carlsbad, CA, USA) to target the coding exon regions of 409 cancer-related genes, which covered the TP53/cell cycle, JAK/STAT, Ras/PI3K, Wnt, receptor tyrosine kinase, chromatin remodelling, DNA repair, TGFβ, and cadherin signaling. We classified gastric cancer patients by tumor based on whether the proportion of altered genes was high or low. The 409 genes (including both oncogenes and tumor suppressor genes) in gastric cancer tumor tissue were sequenced (Appendix Table A1).

### 4.7. Statistical Analysis

All data were analyzed using SPSS version 25 (Armonk, NY, USA). Categorical variables were compared between groups using the chi-square or Fisher’s exact test, and continuous variables were compared using non-parametric Mann-Whitney U test. Univariate and multivariate logistic regression with stepwise procedure was used to identify the independent imaging predictors of CIN subtype gastric cancer. The tumor diameter was dichotomized by cutoff values obtained from a ROC analysis. AUC were calculated to evaluate diagnostic accuracy of each imaging predictor. Two-tailed *p* < 0.05 was considered statistically significant.

## 5. Conclusions

This pilot study of radiogenomic analysis revealed that CT imaging traits may noninvasively predict the TCGA subtype of gastric cancer. In our study, the acute tumor transition angle is the most accurate predictor of the CIN status of gastric cancer, which may provide a preliminary roadmap for personalized medicine.

## Figures and Tables

**Table 1:** Clinical and histopathologic data of training and validation cohorts.

Variable	Training Cohort (n = 40)	Validation Cohort (n = 18)	*p* Value
Age (years), median (range)	68 (36–79)	68 (47–87)	0.69
Male gender	35/40	11/18	0.02
Diameter (cm), median (range)	5.4 (2.8–12.5)	3.7 (1.7–11.6)	0.01
T stage			
1	0	1	0.37
2	1	1	
3	22	5	
4	17	11	
N stage			
0	7	2	0.08
1	8	2	
2	12	3	
3	13	11	
M stage			
0	38	16	0.40
1	2	2	

**Table 2:** Univariate and multivariate logistic regression analysis of predictors of chromosomal instability subtype gastric cancer.

Variables	Univariate			Multivariate		
	OR	95% CI	*p* Value	OR	95% CI	*p* Value
Tumor diameter (cm)	0.69	0.48–1.00	0.051	0.54	0.32–0.90	0.017
Tumor thickness (cm)	3.18	0.92–10.94	0.066			
Location: region						
Cardia, fundus	8.00	0.81–78.83	0.075			
Body	0.50	0.09–2.89	0.438			
Antrum, pylorus	Ref					
Location: curvature						
Lesser curvature	2.11	0.43–10.42	0.359			
Greater curvature	0.44	0.05–4.37	0.487			
Both curvatures	Ref					
Location: wall						
Anterior wall	Ref					
Posterior wall	1.20	0.17–8.66	0.857			
Both walls	1.40	0.28–6.98	0.681			
Tumor margin						
Well-defined	2.33	0.54–10.10	0.257			
Ill-defined	Ref					
Tumor transition angle						
Obtuse angle	Ref			Ref		
Acute angle	7.50	1.53–36.71	0.013	7.41	1.04–52.65	0.045
Tumor shape						
Infiltrative	Ref					
Ulcerated	0.38	0.02–7.00	0.511			
Fungating	1.69	0.28–10.17	0.568			
Polypoid	1.50	0.14–16.27	0.739			
Circumscription						
0–90°	>999.99	<0.01 to >999.99	0.999			
91–180°	0.21	0.04–1.18	0.076			
181–270°	2.25	0.20–25.37	0.512			
271–360°	Ref					
Luminal obstruction						
Presence	1.56	0.35–6.88	0.560			
Absence	Ref					
Serosal invasion						
Presence	Ref					
Absence	2.00	0.47–8.56	0.350			
Enhancement heterogeneity						
Mild	1.00	0.14–7.10	1.000			
Moderate	1.00	0.20–4.96	1.000			
Severe	Ref					
Double-layered enhancement						
Presence	Ref					
Absence	1.35	0.29–6.32	0.702			
Tumor necrosis						
0%–25%	3.00	0.17–54.57	0.458			
26%–50%	4.00	0.17–95.76	0.392			
51%–75%	Ref					
Enlarged lymph node						
Presence	Ref					
Absence	1.71	0.30–9.72	0.543			
Distant metastasis						
Presence	>999.99	<0.01 to >999.99	1.000			
Absence	Ref					

Note—OR, odds ratio; CI, confidence interval.

**Table 3:** Diagnostic accuracy of the imaging predictors of chromosomal instability subtype gastric cancer.

	Sensitivity	Specificity	PPV	NPV	Accuracy	AUC
Training cohort (n = 40)						
Acute tumor transition angle	83.3 (65.3–94.4)	60.0 (26.2–87.8)	86.2 (68.3–96.1)	54.5 (23.4–83.3)	77.5 (61.5–89.2)	0.72 (0.52–0.92)
Tumor diameter ≤7.2 cm	80.0 (61.4–92.3)	60.0 (26.2–87.8)	85.7 (67.3–96.0)	50.0 (21.1–78.9)	75.0 (58.8–87.3)	0.70 (0.50–0.90)
Validation cohort (n = 18)						
Acute tumor transition angle	88.9 (51.8–99.7)	88.9 (51.8–99.7)	88.9 (65.3–98.6)	88.9 (51.8–99.7)	88.9 (51.8–99.7)	0.89 (0.72–1.00)
Tumor diameter ≤7.2 cm	100 (66.4–100)	33.3 (7.5–70.1)	60.0 (32.3–83.7)	100 (29.2–100)	66.7 (41.0–86.7)	0.67 (0.41–0.93)

Note—Data in parentheses are 95% confidence intervals; PPV, positive predictive value; NPV, negative predictive value; AUC, areas under the receiver operating characteristics curve.

**Table 4:** Definition of imaging traits.

Category	Trait Name	Trait Description	Value
Size	Tumor diameter	The largest diameter of the tumor measured on MPR images (cm)	Quantitative
	Tumor thickness	The maximal thickness of the tumor measured on MPR images (cm)	Quantitative
Location	Region	Tumor involvement of the cardia, fundus, body, antrum or pylorus	Ordinal
	Curvature	Tumor involvement of the greater curvature, lesser curvature, or both	Ordinal
	Wall	Tumor involvement of the anterior wall, posterior wall, or both	Ordinal
Morphology	Tumor margin	Tumor margin as well- or ill-defined	Binary
	Tumor transition angle	Transition angle between the tumor and the adjacent normal gastric wall defined as acute or obtuse angle	Binary
	Tumor shape	Tumor shape as infiltrative, ulcerated, fungating, or polypoid	Ordinal
Tumor extent	Circumscription	Circumferential involvement of the tumor as 0–90°, 91–180°, 181–270°, or 271–360°	Ordinal
	Luminal obstruction	Presence or absence of luminal obstruction	Binary
	Serosal invasion	Presence or absence of serosal invasion	Binary
Contrast enhancement	Enhancement heterogeneity	Heterogeneity of contrast enhancement defined as mild, moderate, or severe on portal venous phase images	Ordinal
	Double-layered enhancement	Presence or absence of double-layered contrast enhancement on arterial or portal venous phase images	Binary
	Tumor necrosis	Extent of tumor necrosis defined as 0%–25%, 26%–50%, 51%–75%, or 76%–100%	Ordinal
Metastasis	Enlarged lymph node	Presence or absence of enlarged regional lymph nodes (>1 cm in short axis diameter)	Binary
	Distant metastasis	Presence or absence of distant metastasis	Binary

Note—MPR, multiplanar reconstruction.

## Abbreviations

AUC	Areas under the receiver operating characteristics curve
CI	Confidence interval
CIN	Chromosomal instability
CT	Computed tomography
EBV	Epstein–Barr virus
OR	Odds ratio
ROC	Receiver operating characteristic curve
TCGA	The Cancer Genome Atlas

## Appendix A

**Table A1:** List of studied 409 oncogenes and tumor suppressor genes.

Gene Names										
*ABL1*	*BIRC2*	*COL1A1*	*ERCC4*	*GDNF*	*JUN*	*MDM4*	*NOTCH4*	*PMS1*	*SDHD*	*TLR4*
*ABL2*	*BIRC3*	*CRBN*	*ERCC5*	*GNA11*	*KAT6A*	*MEN1*	*NPM1*	*PMS2*	*9-Sep*	*TLX1*
*ACVR2A*	*BIRC5*	*CREB1*	*ERG*	*GNAQ*	*KAT6B*	*MET*	*NRAS*	*POT1*	*SETD2*	*TNFAIP3*
*ADAMTS20*	*BLM*	*CREBBP*	*ESR1*	*GNAS*	*KDM5C*	*MITF*	*NSD1*	*POU5F1*	*SF3B1*	*TNFRSF14*
*AFF1*	*BLNK*	*CRKL*	*ETS1*	*GPR124*	*KDM6A*	*MLH1*	*NTRK1*	*PPARG*	*SGK1*	*TNK2*
*AFF3*	*BMPR1A*	*CRTC1*	*ETV1*	*GRM8*	*KDR*	*MLLT10*	*NTRK3*	*PPP2R1A*	*SH2D1A*	*TOP1*
*AKAP9*	*BRAF*	*CSF1R*	*ETV4*	*GUCY1A2*	*KEAP1*	*MMP2*	*NUMA1*	*PRDM1*	*SMAD2*	*TP53*
*AKT1*	*BRD3*	*CSMD3*	*EXT1*	*HCAR1*	*KIT*	*MN1*	*NUP214*	*PRKAR1A*	*SMAD4*	*TPR*
*AKT2*	*BRIP1*	*CTNNA1*	*EXT2*	*HIF1A*	*KLF6*	*MPL*	*NUP98*	*PRKDC*	*SMARCA4*	*TRIM24*
*AKT3*	*BTK*	*CTNNB1*	*EZH2*	*HLF*	*KMT2A*	*MRE11A*	*PAK3*	*PSIP1*	*SMARCB1*	*TRIM33*
*ALK*	*BUB1B*	*CYLD*	*FANCA*	*HNF1A*	*KMT2C*	*MSH2*	*PALB2*	*PTCH1*	*SMO*	*TRIP11*
*AMER1*	*CARD11*	*CYP2C19*	*FANCC*	*HOOK3*	*KMT2D*	*MSH6*	*PARP1*	*PTEN*	*SMUG1*	*TRRAP*
*APC*	*CASC5*	*CYP2D6*	*FANCD2*	*HRAS*	*KRAS*	*MTOR*	*PAX3*	*PTGS2*	*SOCS1*	*TSC1*
*AR*	*CBL*	*DAXX*	*FANCF*	*HSP90AA1*	*LAMP1*	*MTR*	*PAX5*	*PTPN11*	*SOX11*	*TSC2*
*ARID1A*	*CCND1*	*DCC*	*FANCG*	*HSP90AB1*	*LCK*	*MTRR*	*PAX7*	*PTPRD*	*SOX2*	*TSHR*
*ARID2*	*CCND2*	*DDB2*	*FAS*	*ICK*	*LIFR*	*MUC1*	*PAX8*	*PTPRT*	*SRC*	*UBR5*
*ARNT*	*CCNE1*	*DDIT3*	*FBXW7*	*IDH1*	*LPHN3*	*MUTYH*	*PBRM1*	*RAD50*	*SSX1*	*UGT1A1*
*ASXL1*	*CD79A*	*DDR2*	*FGFR1*	*IDH2*	*LPP*	*MYB*	*PBX1*	*RAF1*	*STK11*	*USP9X*
*ATF1*	*CD79B*	*DEK*	*FGFR2*	*IGF1R*	*LRP1B*	*MYC*	*PDE4DIP*	*RALGDS*	*STK36*	*VHL*
*ATM*	*CDC73*	*DICER1*	*FGFR3*	*IGF2*	*LTF*	*MYCL*	*PDGFB*	*RARA*	*SUFU*	*WAS*
*ATR*	*CDH1*	*DNMT3A*	*FGFR4*	*IGF2R*	*LTK*	*MYCN*	*PDGFRA*	*RB1*	*SYK*	*WHSC1*
*ATRX*	*CDH11*	*DPYD*	*FH*	*IKBKB*	*MAF*	*MYD88*	*PDGFRB*	*RECQL4*	*SYNE1*	*WRN*
*AURKA*	*CDH2*	*DST*	*FLCN*	*IKBKE*	*MAFB*	*MYH11*	*PER1*	*REL*	*TAF1*	*WT1*
*AURKB*	*CDH20*	*EGFR*	*FLI1*	*IKZF1*	*MAGEA1*	*MYH9*	*PGAP3*	*RET*	*TAF1L*	*XPA*
*AURKC*	*CDH5*	*EML4*	*FLT1*	*IL2*	*MAGI1*	*NBN*	*PHOX2B*	*RHOH*	*TAL1*	*XPC*
*AXL*	*CDK12*	*EP300*	*FLT3*	*IL21R*	*MALT1*	*NCOA1*	*PIK3C2B*	*RNASEL*	*TBX22*	*XPO1*
*BAI3*	*CDK4*	*EP400*	*FLT4*	*IL6ST*	*MAML2*	*NCOA2*	*PIK3CA*	*RNF2*	*TCF12*	*XRCC2*
*BAP1*	*CDK6*	*EPHA3*	*FN1*	*IL7R*	*MAP2K1*	*NCOA4*	*PIK3CB*	*RNF213*	*TCF3*	*ZNF384*
*BCL10*	*CDK8*	*EPHA7*	*FOXL2*	*ING4*	*MAP2K2*	*NF1*	*PIK3CD*	*ROS1*	*TCF7L1*	*ZNF521*
*BCL11A*	*CDKN2A*	*EPHB1*	*FOXO1*	*IRF4*	*MAP2K4*	*NF2*	*PIK3CG*	*RPS6KA2*	*TCF7L2*	
*BCL11B*	*CDKN2B*	*EPHB4*	*FOXO3*	*IRS2*	*MAP3K7*	*NFE2L2*	*PIK3R1*	*RRM1*	*TCL1A*	
*BCL2*	*CDKN2C*	*EPHB6*	*FOXP1*	*ITGA10*	*MAPK1*	*NFKB1*	*PIK3R2*	*RUNX1*	*TET1*	
*BCL2L1*	*CEBPA*	*ERBB2*	*FOXP4*	*ITGA9*	*MAPK8*	*NFKB2*	*PIM1*	*RUNX1T1*	*TET2*	
*BCL2L2*	*CHEK1*	*ERBB3*	*FZR1*	*ITGB2*	*MARK1*	*NIN*	*PKHD1*	*SAMD9*	*TFE3*	
*BCL3*	*CHEK2*	*ERBB4*	*G6PD*	*ITGB3*	*MARK4*	*NKX2-1*	*PLAG1*	*SBDS*	*TGFBR2*	
*BCL6*	*CIC*	*ERCC1*	*GATA1*	*JAK1*	*MBD1*	*NLRP1*	*PLCG1*	*SDHA*	*TGM7*	
*BCL9*	*CKS1B*	*ERCC2*	*GATA2*	*JAK2*	*MCL1*	*NOTCH1*	*PLEKHG5*	*SDHB*	*THBS1*	
*BCR*	*CMPK1*	*ERCC3*	*GATA3*	*JAK3*	*MDM2*	*NOTCH2*	*PML*	*SDHC*	*TIMP3*	
